# Access to affordable daycare and women’s mental health in Rajasthan, India: Evidence from a cluster-randomised social intervention

**DOI:** 10.7189/jogh.14.04063

**Published:** 2024-11-08

**Authors:** Arijit Nandi, Parul Agarwal, Anoushaka Chandrashekar, Shannon Maloney, Robin Richardson, Laxmi Thakur, Sam Harper

**Affiliations:** 1 Institute for Health and Social Policy and Department of Epidemiology, Biostatistics, and Occupational Health, McGill University, Montreal, Quebec, Canada; 2 Institute for Financial Management and Research, Leveraging Evidence for Access and Development, Chennai, Tamil Nadu, India; 3 Department of Health Promotion, Social and Behavioral Health, College of Public Health, University of Nebraska Medical Center, Omaha, Nebraska, USA; 4 Department of Epidemiology, Rollins School of Public Health, Emory University, Atlanta, Georgia, USA; 5 Seva Mandir, Udaipur, Rajasthan, India

## Abstract

**Background:**

Women in India are often responsible for unpaid household work, family caregiving, and paid work, which can contribute to poorer mental health. The provision of childcare has the potential to improve women’s mental health, but evidence on the effects of providing access to daycare is limited.

**Methods:**

We designed a cluster-randomised trial and used data from a sample of 2858 mothers with age-eligible children from 160 village hamlets in rural Rajasthan, India, to evaluate the impact of providing access to a community-based daycare programme on social and emotional aspects of women’s mental health. We conducted a baseline survey in early 2016, randomised hamlets to intervention or control groups approximately six months later, and delivered the final post-intervention survey approximately two years thereafter.

**Results:**

Treatment assignment increased the probability that a respondent used a daycare over the two-year follow-up by 40.9 percentage points. Providing randomised access to a daycare resulted in 0.2 (95% confidence interval (CI) = −0.1, 0.4) fewer symptoms of mental distress, representing a 9.5% decline compared to the baseline mean of 2.1 symptoms, as well as a 3.7 (95% CI = −0.8, 8.3) percentage point increase in the proportion of women who reported feeling very happy, equivalent to an 11.0% increase relative to the baseline mean of 33.6%. Among social indicators, treatment assignment was associated with a 5.6 (95% CI = −1.2, 12.4) percentage point increase in membership in an association, a relative increase of 43.4% compared to the baseline mean of 12.9%. The intervention did not have an appreciable impact on measures of life satisfaction or trust in institutions. Two-stage least squares instrumental variable analyses showed that daycare use decreased mental distress by 0.4 (95% CI = −0.1, 0.8) symptoms, increased the proportion of women who were very happy by 9.4 (95% CI = 0.0, 17.6) percentage points, and increased membership in an organisation by 15.9 (95% CI = 8.4, 23.7) percentage points.

**Conclusions:**

The provision of affordable, community-based daycare was associated with substantial uptake and showed potential for improving mothers’ mental health in a rural context where most women were not employed in the formal labour force.

**Registration:**

ISRCTN clinical trial registry (ISRCTN45369145), registered on 16 May 2016; American Economic Association’s registry for randomised controlled trials (AEARCTR-0000774), registered on 15 July 2015.

Mental health has been defined as ‘the capacities of each and all of us to feel, think, and act in ways that enable us to value and engage in life’ [1]. It is related, but not equivalent to the absence of mental disorders, or to positive indicators of well-being such as happiness. Mental health, therefore, ‘enables positive states of well-being and provides the capacity for people to achieve their full potential’ [2], whereby mentally ‘healthy’ individuals experience a range of human emotions, including distress and sadness [1,3].

Women experience a greater risk of developing mood and anxiety disorders compared to men [4], with global meta-analyses suggesting that, in their lifetime, roughly one in three women have experienced a common mental disorder – a category comprising different types of depression and anxiety – with one in five experiencing it in the past 12 months [5]. Conceptually, gender and other sociodemographic disparities in mental health are related to differential exposure to social and environmental determinants, which interact with genetic, biological, and psychological factors over the life course [2]. As mental health has broad implications for health and development, it is considered an essential component for achieving sustainable socioeconomic development [2].

An estimated 200 million people in India experienced a mental disorder in 2017, with women at greater risk of the most prevalent common mental disorders [6,7]. Few studies have described gender differences in other aspects of emotional well-being in India, including happiness and life satisfaction. One study of urban adults in India found that women reported higher life satisfaction than men in early adulthood, but unlike men, experienced no substantive increase in satisfaction as they aged into older adulthood [8].

Qualitative research from India suggests that women’s experiences and perceptions of depression are shaped by their social environment, including a broader patriarchal sociocultural context that restricts women’s autonomy. This is manifested through stated preferences for male children, limited access to educational opportunities, susceptibility to early and forced marriage, and higher risks of poverty and socioeconomic hardship [9]. Women in India are also more likely to experience acute life stressors, including discrimination and interpersonal violence, and have fewer social resources for coping with social marginalisation [9–12]. They are also often primarily responsible for unpaid household work and family caregiving, as time surveys suggest women spend nearly six hours a day in unpaid domestic work compared to roughly one hour spent by men [13], often by compulsion rather than choice [14]. Women commonly report multitasking, for example, by caring for children while doing other household tasks [15,16], and retain responsibilities for unpaid domestic work even if they take up employment. The constant balancing of these arrangements may increase psychological distress, often expressed as ‘tension,’ and exacerbate women’s risk of prolonged episodes of depression [9,17–19]. Prior research has linked higher levels of housework with poorer mental health [20].

Our understanding of gender disadvantage as a social determinant of mental health in low- and middle-income countries (LMICs) is relatively limited [21], since few studies have integrated gender as a social construct [22]. The provision of affordable and accessible childcare could improve women’s mental health and emotional well-being by reducing role strain and attendant levels of stress [15]. Additionally, access to reliable daycare options might improve women’s mental health by creating space for leisure, participation in the community, and paid work, which could enhance their access to social support, control over household resources, and their overall agency [23,24]. However, in contrast to growing research on the impact of poverty alleviation programmes, few studies have evaluated the impact of access to daycare on women’s mental health and emotional well-being [21].

Here we present the results of a cluster-randomised trial of the impact of providing access to an affordable, community-based daycare programme operated by the non-governmental organisation Seva Mandir on mental health among women living in rural Rajasthan, India. This study extends a prior analysis of the short-term effects of providing access to this programme on levels of women’s mental distress [24] by examining a broader set of outcomes measuring emotional and social aspects of mental health, including levels of general mental distress, happiness, and life satisfaction, as well as membership in associations and trust in institutions; extending the length of follow-up by an additional year; and estimating the effects of maternal exposure (i.e. use of daycare), *vis-à-vis* randomised access to daycare by using an instrumental variable approach.

## METHODS

### Study design and sample

**Figure 1:**
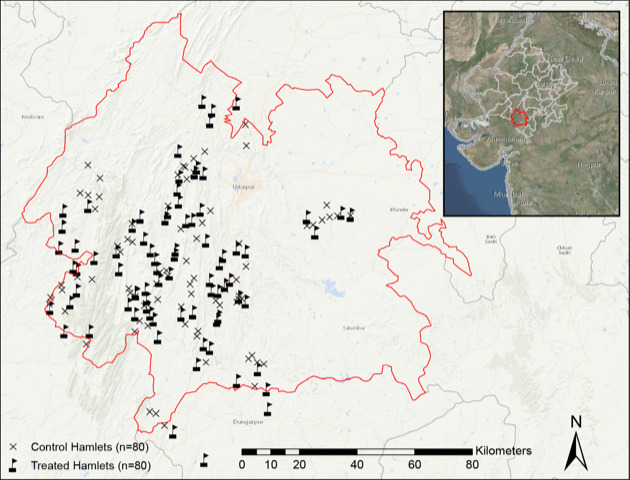
Location of study hamlets in Udaipur district, Rajasthan, India.

**Figure 2:**
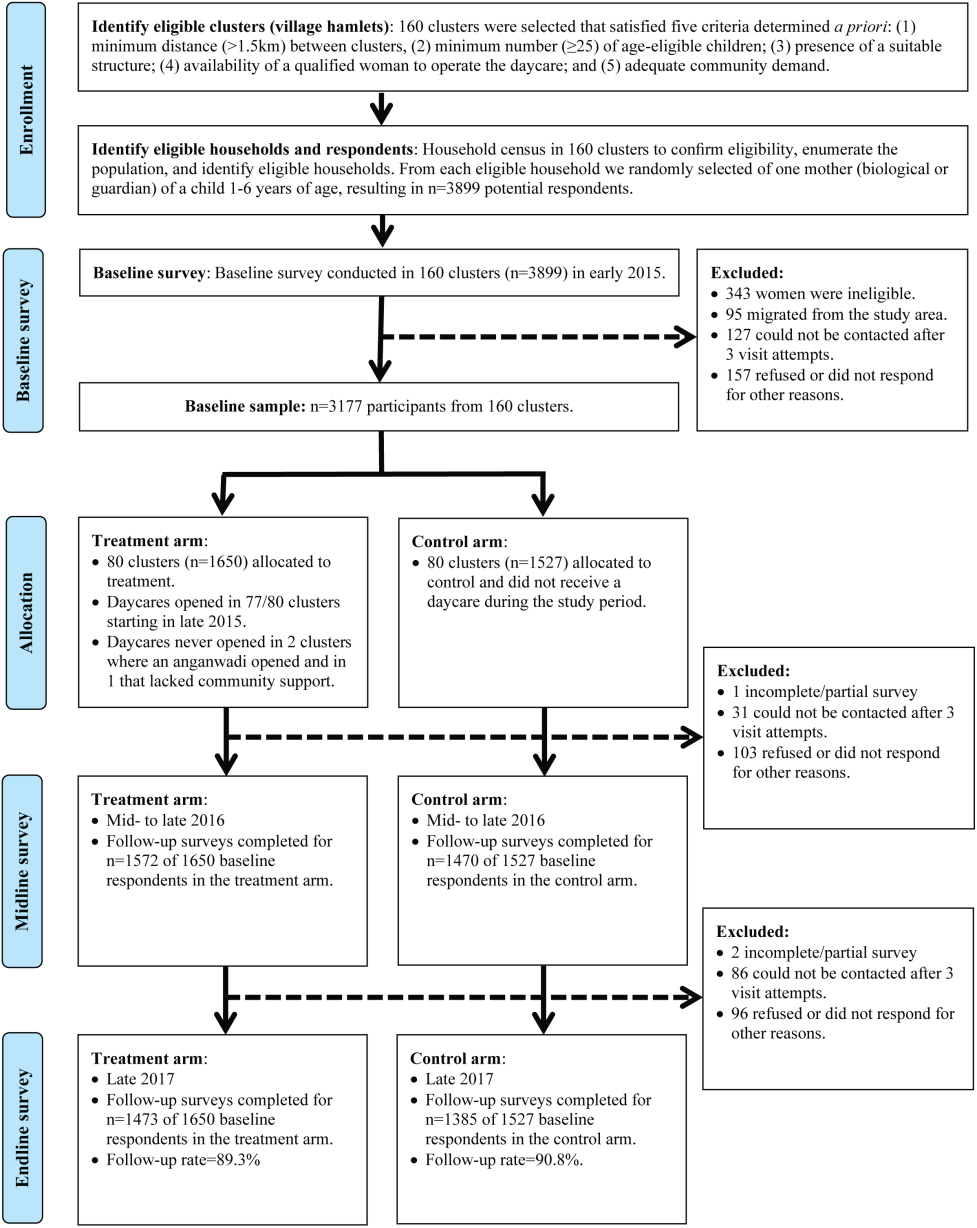
Participant flow diagram.

We conducted this cluster-randomised trial in village hamlets – small, independent settlements that surround a village centre – in five blocks (Badgaon, Girwa, Jhadol, Kherwara, Kotra) in the Udaipur district of Rajasthan, India, where Seva Mandir had not previously established daycares known locally as *balwadis* (**Figure 1**). We first carried out a household census in the five blocks in late 2014 to conﬁrm the eligibility of hamlets, enumerate their population, and identify potential respondents for inclusion. We identified 160 hamlets that satisfied the following pre-determined inclusion criteria: lack of an accessible daycare within 1.5 km to reduce the potential for contamination effects; a minimum number of children (≥25) in the eligible age range of one to six years old to increase demand; an existing structure suitable for a daycare; a qualified woman living in the study hamlet or nearby who would operate the daycare; and adequate demand from the village council (Village Development Committee of Seva Mandir) for a new daycare. Seva Mandir uses these same criteria when establishing a *balwadi*. Next, we conducted a baseline survey within these 160 hamlets in early 2015 among 3177 mothers with a child between one and six years of age, which we had randomly selected from each household. Approximately six months later in late 2015, Seva Mandir offered 80 randomly selected hamlets the opportunity to open a *balwadi*, with the remaining 80 hamlets serving as the control group and being ineligible to receive a *balwadi* until data collection was completed. We delivered the first post-intervention follow-up survey in mid to late 2016 among 3042 respondents, followed by a second follow-up survey of 2858 of the 3177 baseline participants (90.0% follow-up rate) approximately one year later in mid to late 2017. Further details on the sample selection are shown in **Figure 2**, with additional information on the study design, including power calculations and study procedures, available in the trial protocol [25].

After we provided the potential participants with a description of the study objectives, procedures, potential risks and benefits, voluntary nature, confidentiality and privacy protections, and compensation, each eligible respondent could refuse or consent to participate, either in written form if they could read and write or orally if they could not. At the completion of each interview, respondents were given a gift to compensate for their time, valued at INR 100. The Institutional Review Board of McGill University’s Faculty of Medicine and the Human Subjects Committee of the Institute for Financial Management and Research in Chennai, India approved this study (A08-E61-14A).

### Randomisation

We conducted a simple, cluster-level randomisation of the 160 eligible hamlets with a 1:1 allocation ratio. Daycare programmes were randomised at the level of the village hamlet since it was not feasible to do so at the level of individual families within communities. We stratified the randomisation of hamlets by block (n = 5) to prevent variations in the distributions of blocks across treatment groups (e.g. if women in control hamlets were more likely to reside in blocks with greater access to social organisations). Hamlets were randomised to either the treatment or control within blocks using a random number generator in Stata, version 17 (StataCorp LLC, College Station, TX, USA). It was not possible to blind study personnel or participants after the implementation of the intervention; however, we concealed the allocation of hamlets to treatment or control status until after the baseline survey to minimise opportunities for bias in the recruitment of participants and the baseline survey. Further details on the randomisation procedure are available in the research protocol [25].

### Intervention

The intervention was the randomised offer of full-time daycare centres in areas where they were not yet available. *Balwadis* are community-based daycares implemented by Seva Mandir, which operates daycare programmes in other areas in the Udaipur district. Care in the *balwadis* is available from 9 am to 4 pm and costs INR 150 per child each year; if a household could not pay this fee, they could provide in-kind support to the daycare centre (e.g. cleaning). In addition to childcare, *balwadis* provide nutritious food and supplements, basic medicines, and preschool education to children in two learning groups (i.e. 1–2 and 3–6 years of age). Additionally, the programme maintains children’s immunisation records and follows up with parents and government nurses, with the aim of increasing immunisation coverage. The *balwadis* are operated by local women called *sanchalikas*, who are hired by Seva Mandir and receive about 20 days of training each year regarding their roles and responsibilities. Parents meet with the *sanchalika* quarterly to discuss their child’s progress. *Balwadis* were established in 77 of the 80 village hamlets randomised to the treatment group.

### Measures

#### Outcomes

Emotional aspects of mental health were measured by symptoms of mental distress, happiness, and life satisfaction [26]. We measured mental distress using the Hindi translation of the General Health Questionnaire (GHQ-12) [27,28], which includes 12 items that ask women about how they have been feeling recently. For example, women were asked, ‘Have you recently felt constantly under strain?,’ with potential responses being ‘not at all,’ ‘no more than usual,’ ‘rather more than usual,’ and ‘much more than usual.’ We scored items using the 0-0-1-1 scoring system often applied in India to dichotomise each symptom as occurring more than usual or not [29,30]. Total scores for the GHQ-12 can range from 0 to 12, with higher scores indicating greater mental distress. Although not a clinical diagnosis of mental illness, a systematic review of validation studies of screening tools for common mental disorders in LMICs concluded that the GHQ-12 performed well in detecting common mental disorders [31]. We measured happiness by asking respondents if ‘taking all things together, would you say you are very happy, rather happy, not very happy, or not happy at all,’ with responses dichotomised as very happy if a woman indicated ‘very happy’ or not very happy if a woman indicated ‘rather happy,’ ‘not very happy,’ or ‘not happy at all’ for analysis. Lastly, we measured life satisfaction by asking respondents ‘all things considered, how satisfied are you with your life as a whole these days?’ on a scale ranging from 1 (‘completely dissatisfied’) to 10 (‘completely satisfied’), as done in the World Values Survey [32].

We measured social aspects of women’s mental health by asking participants about their membership in associations and trust in public institutions, which have been related to mental health in prior research [33]. Specifically, we constructed a binary indicator for membership in an association by asking respondents if they were members of any association, group, or club which holds regular meetings. Trust in institutions was measured by asking respondents about their level of confidence, ranging from 1 (‘no confidence’) to 10 (‘a great deal of confidence’), in each of the following: politicians, government, *panchayat* (village council), non-governmental organisations, bank and insurance agencies, and police. We used a polychoric principal component analysis to derive a summary trust score of these six ordinal variables; we retained the first component based on a Scree plot of eigenvalues, which explained 44% and 47% of the shared variance at baseline and endline, respectively [34,35].

#### Covariates

Covariates included the baseline values of the outcomes described above. We compared the distribution of baseline sociodemographic characteristics across treatment groups, including: age in years; whether the respondent ever attended school or not; whether the respondent was currently married or not married, including widowed, divorced, separated, or living together; the age at marriage, if married; whether the respondent’s husband ever attended school; whether the household had a Below the Poverty Line card or not; the number of children under age 18 years who were living at home; Hindu religion or other, including other religion, no religion, or unknown; the time spent in the past day caring for young children; whether the respondent worked in the past seven days or not; whether respondents who reported working in the past 12 months were self-employed or not, including working for a family member, the government, or someone else; whether respondents who reported working in the past 12 months worked year round or not, including seasonally/part of the year or once in a while; whether respondents who reported working in the past 12 months were paid in cash or not; whether the respondent reported spending any time in the past day on paid work; total income received by the household over the past year in INR. We included block as a covariate because it was used to stratify the randomisation.

#### Statistical analysis

We estimated the impact of randomised assignment of the treatment, i.e. access to a *balwadi*, using an intent-to-treat (ITT) analysis with adjustment for stratification by block. We used a logistic regression model for binary outcomes (very happy vs not very happy, membership in any association vs no membership):



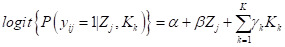



Here, *y_ij_* is the outcome of interest for an individual *i* in cluster *j*, *Z_j_* is the main cluster-level treatment assignment variable (1 for intervention, 0 for control), and *γ_k_* are coefficients for each of the *K* blocks on which we stratified randomised treatment assignment. The coefficient β is our primary estimate of interest and we reported marginal effects on the absolute risk scale with corresponding 95% confidence intervals (CIs).

To improve precision, we added baseline covariates to the first equation that are strong predictors of each outcome (specifically, pre-treatment values of the outcome measured at baseline), since this can lead to meaningful increases in power [36,37]:



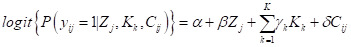



Here, *C_ij_* is the value of outcome *y_ij_* at baseline. We used negative binomial and linear regression models, respectively, for count (symptoms of mental distress) and continuous (life satisfaction scale, trust in institutions) outcomes. To account for non-independence of errors among observations in the same hamlet, we used cluster-robust variance estimators in all models [38,39].

Since not all women in treated clusters accessed daycare, in addition to the ITT analyses of the impact of treatment assignment, we also estimated the effect of maternal use of a *balwadi* among women who availed themselves of services due to treatment assignment. We estimated this effect, known as the complier average causal effect, using two-stage least squares instrumental variables analyses. The core assumptions for these models are: random assignment of hamlets increases maternal exposure or use of daycare; random assignment to daycare affects outcomes only through its impact on the actual take-up and use of daycare; and absence of any within-cluster spillover effects [40]. In the ﬁrst stage equation, we estimated the impact of randomised treatment assignment on daycare use:



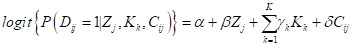



Here, *D_ij_* represents whether the respondent reported any *balwadi* use during follow-up. We used the coefficients from this equation to predict the probability of using a *balwadi*, *D̂_ij_*, as well as the residual, *r̂_ij_* = *D_ij_* − *D̂_ij_*, which we then added to the second stage equation along with the indicator for treatment status, shown here for a binary outcome [41]:



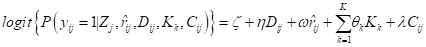



Here, *y_ij_* is the outcome of interest, *r̂_ij_* is the residual calculated from the third equation, and the coefficient η is used to generate the marginal complier average causal effect; including the residual rather than the predicted value of the treatment from the first stage is less biased for binary outcomes [42]. As in our ITT analyses, we used negative binomial and linear regression models in this equation for count and continuous outcomes. We estimated cluster-robust standard errors in both equations and obtained standard errors for instrumental variable estimates using bootstrapping with 1000 replications [43,44].

## RESULTS

### Descriptive findings

Baseline characteristics were balanced between the treatment and control arms of the trial (**Table 1**). One-quarter of women had ever attended school compared to 65.3% of husbands, and the mean age at marriage for women was 17.5 years. Women had 3.3 children on average and reported spending approximately 2.5 hours caring for children in the past 24 hours. Nearly two-thirds of women worked in the past week; however, only 9% of respondents who worked in the past year were compensated in cash. The mean household income received over the past year was INR 53 806 and one-half of households reported having a Below the Poverty Line card. The women had an average of 2.1 symptoms of mental distress, 33.6% of respondents reported feeling very happy, the average life satisfaction score was 6.0, and 12.9% of respondents were members of an association, group, or club that holds regular meetings.

**Table 1:** Baseline characteristics for the total sample and stratified by treatment status*

Variable	Sample size	Total sample	Control	Treated
Age	2856	30.0 (6.8)	30.1 (6.7)	30.0 (6.8)
Any schooling	2857	25.6 (43.6)	26.4 (44.1)	24.7 (43.1)
Currently married	2858	98.6 (11.9)	98.8 (10.7)	98.3 (12.9)
Age at marriage	2766	17.5 (2.8)	17.5 (2.7)	17.4 (3.0)
Any schooling (husband)	2835	65.3 (47.6)	68.1 (46.6)	62.6 (48.4)
Below poverty line	2849	50.4 (50.0)	51.2 (50.0)	49.6 (50.0)
Number of children	2858	3.3 (1.6)	3.3 (1.6)	3.3 (1.6)
Hindu religion	2857	71.8 (45.0)	72.3 (44.8)	71.3 (45.3)
Minutes on childcare (direct)	2854	148.1 (140.6)	150.7 (140.4)	145.5 (140.9)
Worked in past seven days	2858	62.0 (48.5)	61.7 (48.6)	62.3 (48.5)
Self employed	2720	37.1 (48.3)	34.2 (47.4)	39.8 (49.0)
Works year round	2720	8.1 (27.3)	9.0 (28.7)	7.3 (26.0)
Paid cash for work	2720	9.0 (28.6)	9.6 (29.5)	8.4 (27.8)
Any time on paid work	2855	5.5 (22.8)	5.5 (22.8)	5.5 (22.8)
Total income (INR)	2857	53 805.9 (42 575.3)	54 352.3 (43 796.6)	53 291.8 (41 401.7)
Symptoms of mental distress	2855	2.1 (2.5)	2.2 (2.5)	2.1 (2.4)
Very happy	2856	33.6 (47.3)	32.0 (46.7)	35.2 (47.8)
Life satisfaction	2768	6.0 (2.6)	6.0 (2.6)	6.1 (2.6)
Trust in institutions (score)	2838	0.0 (1.6)	0.0 (1.6)	0.0 (1.7)
Membership in associations	2855	12.9 (33.6)	11.8 (32.2)	14.0 (34.7)

*Distributions presented as mean (SD).

### Intent-to-treat estimates for the impact of random assignment to a *balwadi*

Treatment assignment decreased the GHQ-12 score by 0.2 (95% CI = −0.1, 0.4) symptoms, representing a roughly 9.5% decline compared to the baseline mean of 2.1 symptoms. Providing access to a *balwadi* increased the proportion of women who reported feeling very happy by 3.7 percentage points (95% CI = −0.8, 8.3), a 11.0% increase compared to the baseline mean. Providing randomised access to a *balwadi* did not appreciably influence respondents’ life satisfaction scores, with randomised access associated with a 0.1 percentage point (95% CI = −0.2, 0.3) reduction in the life satisfaction score. Random assignment did not affect trust in institutions, which declined by 0.1 points (95% CI = −0.1, 0.3) in the treatment compared to the control group. However, membership in an association increased by 5.6 percentage points (95% CI = −1.2, 12.4) among those randomised to the treatment compared to the control group, a relative increase of 43.4% (**Table 2**).

**Table 2:** Estimated impacts of providing access to a *balwadi* on women’s mental health from intent-to-treat analyses

	Without baseline controls		Controls for baseline value	
**Variables**	**Sample size**	Estimate (95% CI)*	**Sample size**	**Estimate (95% CI)**
Symptoms of mental distress	2858	−0.2 (−0.4, 0.1)	2855	−0.2 (−0.4, 0.1)
Very happy	2858	3.9 (−0.7, 8.4)	2856	3.7 (−0.8, 8.3)
Life satisfaction	2856	0.0 (−0.3, 0.2)	2766	−0.1 (−0.3, 0.2)
Trust in institutions	2858	−0.1 (−0.3, 0.1)	2838	−0.1 (−0.3, 0.1)
Member of an association	2858	6.1 (−1.0, 13.2)	2855	5.6 (−1.2, 12.4)

CI – confidence interval

*Estimates are average treatment effects from intent-to-treat treatment analyses reported on the additive scale.

### Instrumental variable estimates for the impact of using a *balwadi*

Randomised access to the programme increased individual exposure or uptake of daycare by 40.9 percentage points. *Balwadi* use was estimated to decrease the GHQ-12 score by 0.4 symptoms (95% CI−0.1, 0.8), a 19.0% reduction relative to the baseline mean. Additionally, *balwadi* use increased the proportion of women who were very happy by 9.4 percentage points (95% CI = 0.0, 17.6) and membership in an organisation by 15.9 percentage points (95% CI = 8.4, 23.7), representing 28.0% and 123.3% increases relative to their baseline values, respectively (**Table 3**).

**Table 3:** The impact of any *balwadi* use on women’s mental health from instrumental variable analyses

Variables	Sample size	Estimate (95% CI)*	F-statistic
Symptoms of mental distress	2855	−0.4 (−0.8, 0.1)	187.2
Very happy	2856	9.4 (0.0, 17.6)	189.1
Life satisfaction	2766	−0.1 (−0.6, 0.3)	182.1
Trust in institutions	2838	−0.1 (−0.4, 0.1)	188.0
Member of an association	2855	15.9 (8.4, 23.7)	188.9

CI – confidence interval

*We derived the estimates from a two-stage model. The first stage regressed maternal *balwadi* use on the randomised treatment indicator and block using cluster robust standard errors. In the second stage, we utilised the predicted probability of *balwadi* use from the first stage to estimate the impact of *balwadi* use on our outcomes of interest using logistic, negative binomial, and linear regression models for binary, count, and continuous outcomes, respectively, and reported marginal effects. We calculated standard errors by bootstrapping this procedure 1000 times.

## DISCUSSION

Our findings suggest that providing access to an affordable, community-based daycare programme in rural Rajasthan, India, was associated with modest improvements in maternal mental health and emotional well-being approximately two years after the initial intervention, including a slight reduction in mental distress and an increase in happiness. However, it is worth noting that these effects were estimated with imprecision. The intervention also led more women to join an association but did not affect trust in institutions. Since the uptake of the daycare programme in treated villages was not universal, we conducted instrumental variable analyses to estimate the effect of daycare uptake, which indicated that the use of a daycare substantially lowered maternal mental distress and increased their happiness and association membership. Population-level interventions designed to address structural determinants of health have the potential to improve women’s mental health and well-being, and to complement efforts to expand access to culturally appropriate mental health care services [45].

Unmet need for mental health services remains a persistent problem in LMICs; India is no exception, as it has roughly 0.3 psychiatrists per 100 000 population [46] and an estimated 15.5% of individuals with any self-reported mental illness were receiving treatment in 2015–16 [47]. Expanded access to mental health care services, for example through task-sharing interventions, may be insufficient for addressing the mental health treatment gap, since distress and expressions of ‘tension’ in South Asia are often related to underlying social or structural factors, and may not respond to clinical interventions designed to treat mental disorders [48].

Evidence on the impact of social interventions on mental health or gender inequalities in mental health in LMICs has focussed primarily on poverty alleviation interventions, including cash transfer and microcredit programmes. Extant evidence is mixed, with some evidence of positive effects on mental health and psychological well-being across genders [49,50]; improvements restricted to boys or adult males [51,52]; improvements restricted to girls or adult females [53]; improvements for young males, but increased depressive symptomatology for young females [54]; or no overall impact [55,56]. As most of these studies are derived from sub-Saharan Africa, the only evidence from India concerns the impact of the ‘Janani Suraksha Yojana’ maternal cash transfer programme, which concluded that receipt of cash payments was associated with reductions in maternal depression, but had no impact on measures of emotional well-being, including happiness [57].

Extant evidence from LMICs finds mixed results on the impact of providing access to daycare on women’s mental health. Using a regression discontinuity design, access to childcare centres was found to increase symptoms of stress and depression among mothers in Ecuador by roughly 0.4 standard deviations [58]. An observational study showed that women with children enrolled in a daycare programme in Mexico reported similar levels of stress and depressive symptoms compared to women on a waitlist for the programme [59]. Observational studies, however, are susceptible to unmeasured confounding, since women who utilise childcare may be different from those who do not in ways that are related to their mental health. In the only randomised controlled trial to measure the impact of daycare on women’s mental health in LMICs, we showed that providing access to the *balwadi* programme in rural Rajasthan, India resulted in a modest 11% decline in symptoms of mental distress measured roughly one year after the intervention [24]. This study, which extended the follow-up period by an additional year, suggests that improvements in mental health persisted roughly two years after the initial rollout of the daycare programme. Furthermore, access to daycare and accompanying use of a *balwadi* were associated with increases in happiness and membership in an association. It is challenging to put the magnitude of these effects in context with prior research in India, since few studies have estimated the effects of social interventions on mental health using comparable survey instruments.

The effects of daycare on women’s mental health may be moderated by whether women were induced to work as a result of the programme, with the daycare programmes in Mexico and Ecuador associated with substantively larger increases in women’s employment compared to the *balwadi* programme, which had a small positive impact on women’s participation in paid work, but not other indicators of labor force participation [58–60]. Potential mechanisms linking access to and use of daycare to improved mental health may be related to spending less time on childcare, reductions in role strain and related stress levels, and opportunities for greater social participation. We observed large increases in membership in associations among women who used a *balwadi* because of receiving randomised access to the programme.

Several limitations should be considered when interpreting these findings. First, approximately 10% of baseline respondents were lost to follow-up during the two-year follow-up period, which may have created a selection bias if, for example, women experiencing greater levels of distress were less likely to participate in follow-up surveys, and these losses were differential by treatment status. Second, non-compliance or imperfect compliance in the form of a *balwadi* failing to open, opening late, closing before the end of the study period, or operating for limited days or hours could dilute treatment effects. According to camera monitoring data from Seva Mandir, the *balwadi*s were open for more than two-thirds of the days they were scheduled to operate. Third, although we used buffers to limit spillovers, approximately 9% of mothers in control villages reported some use of a *balwadi* during follow-up, which may have attenuated the ITT estimates. Fourth, the instrumental variable analyses assume that randomised access to a *balwadi* affects mental health through maternal *balwadi* use. However, the use of a *balwadi* by neighbors or kin may have affected the respondent’s mental health, irrespectively of their own use. Fifth, we did not collect information on the use of mental health services or treatment for mental distress. However, access to services is very limited in the study context and we would not expect imbalances in treatment across the intervention and control groups.

## CONCLUSIONS

The findings from this cluster-randomised trial suggest that access to and use of daycare had positive effects on women’s mental health in rural Rajasthan, India, including a reduction in mental distress and an increase in happiness, as well as a substantial increase in membership in an association. Our study shows promising evidence that, in similar environments, providing access to affordable daycare could improve women’s mental. Further research is needed to evaluate the impact of daycare access on women’s mental health in settings with diverse labour markets and employment opportunities.
